# Outcomes for Step-Wise Implementation of a Human Papillomavirus Testing–Based Cervical Screen-and-Treat Program in El Salvador

**DOI:** 10.1200/GO.20.00206

**Published:** 2020-10-16

**Authors:** Karla Alfaro, Mauricio Maza, Juan C. Felix, Julia C. Gage, Philip E. Castle, Todd A. Alonzo, Andrea Chacón, Enrique González, Montserrat Soler, Gabriel Conzuelo-Rodriguez, Rachel Masch, Miriam Cremer

**Affiliations:** ^1^ Basic Health International, San Salvador, El Salvador and New York, NY; ^2^ Department of Pathology, Medical College of Wisconsin, Milwaukee, WI; ^3^ Division of Cancer Epidemiology and Genetics, National Cancer Institute, Rockville, MD; ^4^ Division of Cancer Prevention and Division of Cancer Epidemiology and Genetics, National Cancer Institute, NIH/DHHS, Rockville, MD; ^5^ Keck School of Medicine, University of Southern California, Los Angeles, CA; ^6^ Unidad de Cáncer, Ministerio de Salud República de El Salvador, San Salvador, El Salvador; ^7^ Ob/Gyn & Women’s Health Institute, Cleveland Clinic, Cleveland, OH

## Abstract

**PURPOSE:**

The Cervical Cancer Prevention in El Salvador (CAPE) project is a public-sector intervention introducing lower-cost human papillomavirus (HPV) testing in all four departments of the Paracentral region that screened a total of 28,015 women. After demonstrating success of an HPV screen-and-treat (S&T) algorithm over colposcopy management in the first two phases, the third phase scaled up the S&T strategy. We present results from phase III and evaluate S&T components across the entire project.

**METHODS:**

During phase III, 17,965 women age 30-59 years underwent HPV testing. HPV-positive women were asked to return and, if eligible, received gas-based cryotherapy. We compare loss to follow-up and time intervals between S&T steps across the three phases.

**RESULTS:**

There were no differences in HPV positivity across phases (phase I, 11.9%; phase II, 11.4%; phase III, 12.3%; *P* = .173). Although most HPV-positive women completed indicated follow-up procedures within 6 months in phases I (93.3%, 111 of 119) and II (92.3%, 429 of 465), this proportion declined to 74.9% (1,659 of 2,214; *P* < .001) in phase III. Mean days between testing and delivery of results to patients increased over program phases (phase I, 23.2 days; phase II, 46.7 days; phase III, 99.8 days; *P* < .001).

**CONCLUSION:**

A public-sector implementation of an HPV-based S&T algorithm was successfully scaled up in El Salvador, albeit with losses in efficiency. After CAPE, the Ministry of Health changed its screening guidelines and procured additional tests to expand the program.

## INTRODUCTION

Although cytology-based cervical cancer screening continues to be widely used, molecular testing of the human papillomavirus (HPV) has emerged as a more sensitive screening tool.^1,2^ In developing countries, low quality of cytology services, lack of adequately trained health providers, and high rates of loss to follow-up have further limited the success of cytology-based screening.^3,4^ Recognizing these constraints and the advantages of molecular HPV testing, the WHO has created guidelines for the use of HPV screening tests for cervical cancer in low-resource settings.^5^ In these guidelines, women who screen HPV positive can bypass colposcopic examination and undergo a visual assessment to determine eligibility for immediate treatment with cryotherapy. The feasibility of such screen-and-treat (S&T) paradigms has been assessed using different combinations of HPV screening and ablative treatment modalities.^6-9^

**CONTEXT**

**Key Objective**
Is it feasible to implement an effective and sustainable cervical cancer screening program in a low- or middle-income country (LMIC)?
**Knowledge Generated**
Combining low-cost human papillomavirus (HPV) testing with a screen-and-treat protocol, Cervical Cancer Prevention in El Salvador (CAPE) is a public sector cervical cancer screening program that was introduced and scaled up in an LMIC. Despite ongoing barriers, such as delays in result delivery and loss to follow-up of women not eligible for screen-and-treat, the program has now been expanded nationally and included in El Salvador’s national cervical cancer control guidelines.
**Relevance**
The program highlighted the importance of collaboration by different stakeholders to maximize the use of existing resources in the country. The CAPE model can be used as a blueprint for other nations to address the burden of cervical cancer incidence and mortality.

Despite the potential of HPV testing, the high cost of these assays has hampered widespread adoption of S&T. Currently, one low-cost HPV test (careHPV, QIAGEN, Gaithersburg, MD) is prequalified by the WHO,^10^ but there are other tests in development.^11,12^ There is a need for evidence of best practices in HPV testing implementation in lower-resource settings. Public-sector HPV screening projects have been successful but have used cytology as a triage test or colposcopy with biopsy to confirm diagnosis before treatment.^13-15^ The feasibility of the HPV test as a screening tool has not been confirmed in regions with limited capacity for cytology and histology.

Here we evaluate outcomes on the basis of the first public-sector S&T intervention using lower-cost HPV testing. The project took place in El Salvador, where cervical cancer mortality is 18.5 per 100,000 women (age adjusted), and the estimated screening rate is one of the lowest in Latin America at 19%.^16,17^ HPV vaccination is not yet available in the public sector. Previous research has shown that adherence to a cytology-based regimen is poor at 44%.^18^ To address this issue, in 2012 the El Salvador Ministry of Health (MOH) submitted a proposal to obtain careHPV tests through the QiagenCares donation program. The nonprofit Basic Health International (BHI) served as technical advisor. The application was successful, and the MOH moved forward with the Cervical Cancer Prevention in El Salvador (CAPE) project.

CAPE was designed as a three-phase demonstration project to compare the feasibility of the national colposcopy management strategy against a primary HPV-based S&T paradigm and to incrementally scale up the most successful strategy. The implementation relied on the existing resources of the national public health system, with BHI providing technical support and training to MOH personnel. In CAPE phases I and II,^19,20^ HPV-positive women assigned to the S&T cohort were more likely to complete treatment within 6 months compared with those assigned to the colposcopy management group (phase I: 98.3% *v* 68.8%, *P* < .001; phase II: 90.8% *v* 65%, *P* < .001).^19,20^

In the S&T algorithm, women were screened with the careHPV test. Returning HPV-positive women underwent visual assessment for treatment (VAT) and, if eligible, cryotherapy treatment on the same day. Women were considered ineligible for cryotherapy if the lesion was > 75% of the cervix, penetrated the endocervical canal, or was suspicious for cancer, or if VAT was unsatisfactory (the transformation zone was not fully visible). Ineligible women were referred to colposcopy, biopsy, and treatment pending pathology findings. Cost-effectiveness analyses used CAPE and epidemiologic data from El Salvador to model long-term health and economic outcomes of S&T and conventional colposcopy management. S&T resulted in a greater reduction of lifetime cervical cancer risk (a difference of > 15%) at a lower cost per woman.^21,22^ In light of this evidence, the MOH made the decision to expand the S&T protocol as the approach with most potential for a national scale-up. Here we present results from CAPE phase III, compare findings from the S&T cohorts across the three phases, and discuss challenges and lessons learned throughout the project.

## METHODS

**FIG 1:**
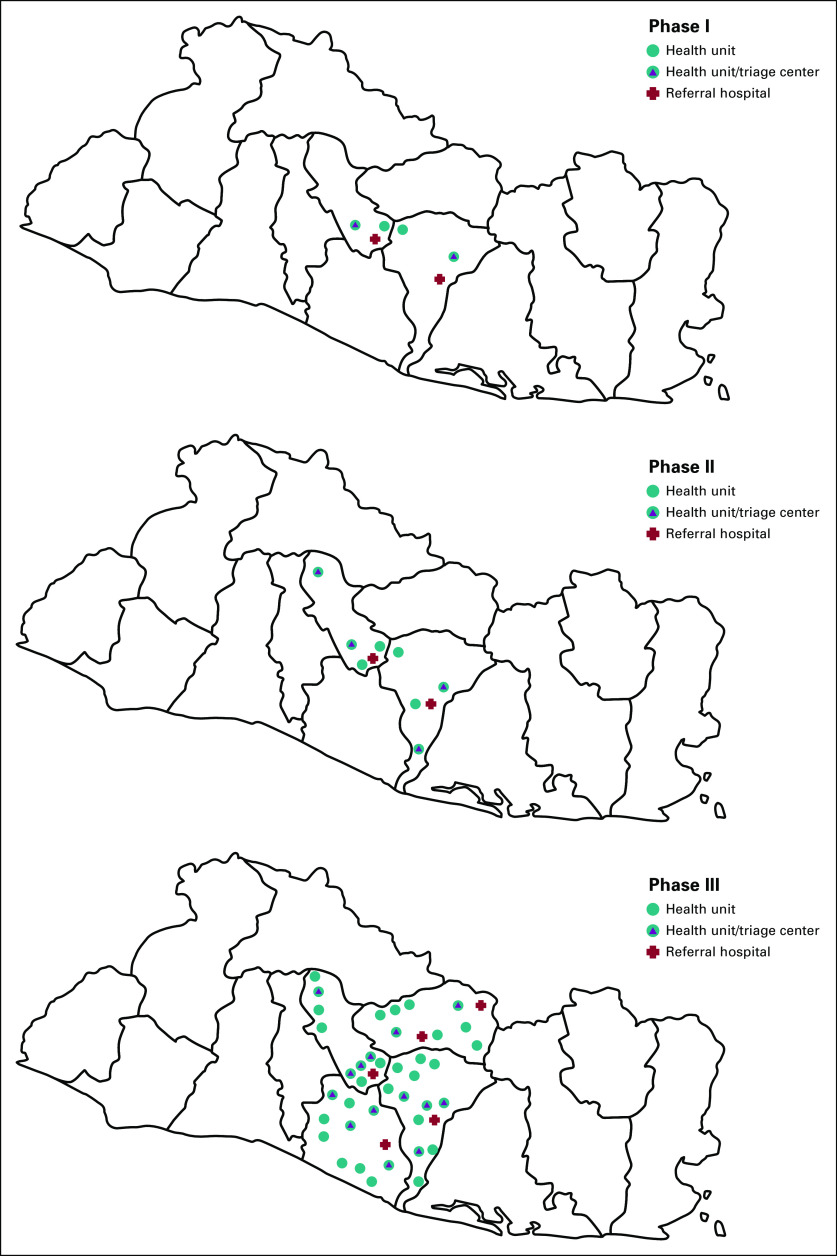
Locations where Cervical Cancer Prevention in El Salvador (CAPE) was implemented in the Paracentral region of El Salvador.

CAPE was implemented primarily in rural areas of the Paracentral region of El Salvador (Fig 1). Because 80% of the country’s population is covered by the public sector,^23^ CAPE was intended to serve to majority of women in target areas. Phase I was carried out from October 2012 to March 2013, and phase II took place between October 2013 and July 2014 (both in the San Vicente and Cuscatlán departments).^19,20^ In phase I, 2,000 women age 30-49 years were enrolled and divided equally into the S&T and colposcopy management cohorts. In phase II, 8,050 women age 30-49 years were enrolled, with 4,087 assigned to S&T and 3,963 assigned to colposcopy management. Because CAPE was designed as a demonstration project rather than a clinical trial, these were nonrandom assignments made by the MOH using the national census to obtain the number of age-eligible women living in the catchment areas of target health units. Units were then assigned to the two cohorts to match the number of women.

**FIG 2:**
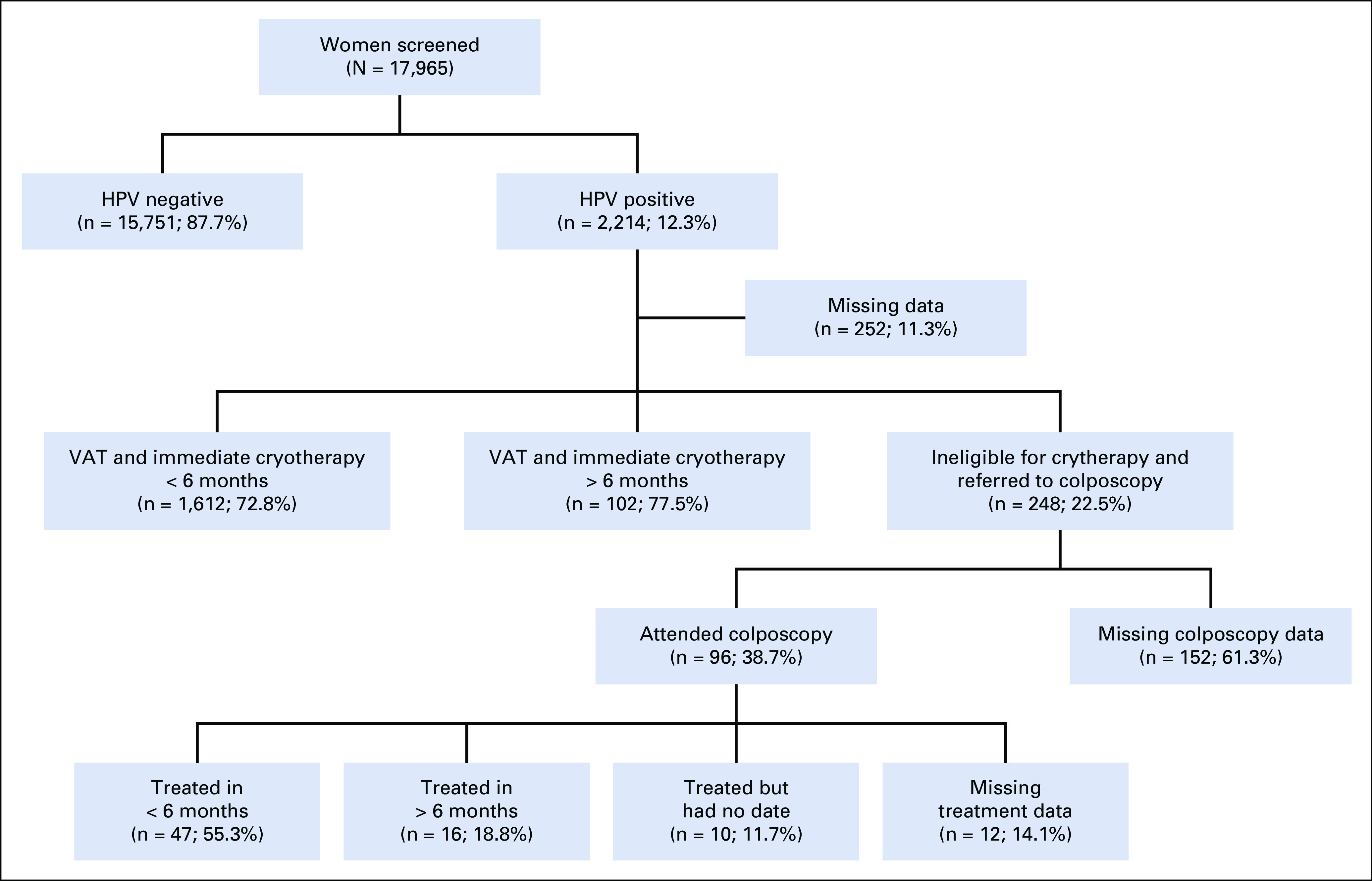
Patient flowchart of phase III of Cervical Cancer Prevention in El Salvador (CAPE).

CAPE phase III (May 2015 to April 2017) enrolled 20,000 women aged 30-59 years living in communities served by 63 health units that were screening locations across all four departments of the Paracentral region (San Vicente, Cuscatlán, La Paz, and Cabañas). HPV-positive women were managed only by S&T. Fourteen clinics were equipped with cryotherapy units to provide treatment of eligible HPV-positive women. These were strategically distributed to minimize transportation barriers. Five regional hospitals with colposcopy facilities were designated as referral locations (Fig 1) for HPV-positive women ineligible for cryotherapy. The MOH increased the age range from 30-49 to 30-59 years to reach more at-risk women. According to local census records, this increased the pool of potential participants from 98,032 to 132,173. A flow chart of the project’s third phase is presented in Figure 2.

Throughout CAPE, training was provided to a total of eight laboratory technicians, 610 community health promoters (CHPs), 360 nurses, and 223 physicians. Before the beginning of phase III, BHI hired and trained five physicians to deliver group trainings to participating MOH doctors, nurses, laboratory technicians, and CHPs. In groups of 30, CHPs attended 2-day workshops that covered the female reproductive system, HPV and cervical cancer risk, and guidance on participant recruitment. Doctors and nurses were also divided into groups of 30 to take part in 1-day training workshops that included the natural history of cervical cancer, HPV testing, treatment options, and program logistics (eg, preparing HPV samples for transportation, delivering test results, and recording patient data).

Fourteen general practitioners were designated by the MOH to perform VAT and gas-based cryotherapy at the designated triage centers. Training included a 1-day theoretical component and a 4-day practicum with patients. To do this, three provisional clinics were set up in a local health unit. Trainees were supervised by two gynecologists. CHPs scheduled 100 women per day age 30-49 years who were either unscreened or due for a screening. For the training, women were screened using visual inspection with acetic acid, a method included in El Salvador’s guidelines at the time. Screen-positive women were assessed for cryotherapy treatment using the VAT criteria of phases I and II. In the subsequent 3 months, an experienced gynecologist conducted monitoring visits at the triage centers to ensure adequate implementation of clinical procedures. BHI personnel monitored program logistics. In phases one and two, four laboratory technicians had been trained in analysis of the HPV tests by staff from the test manufacturer; four technicians were added in phase III.

Recruitment of participants was conducted by CHPs, who live in the same communities where they work. CHPs are responsible for monitoring and recording the participation of 200-500 families in primary health care programs. CHPs used their records to identify and contact, through phone calls or home visits, age-eligible women who were under-screened, never screened, or due for screening. If a woman agreed, she was scheduled to attend her local health unit within 15 days to undergo HPV testing. Appointments were organized in groups of 10-20 women for the same day. Before testing, the health unit physician or nurse delivered a cervical cancer educational session to the women and explained follow-up procedures for women with positive results. Test samples were collected by the health unit staff and transported to four MOH laboratories for refrigerated storage at 8°C, as per the manufacturer’s instructions, and eventual analyses.

All women obtained an appointment to receive their results at the health unit in 30 days. Test samples can be run in 4-6 hours but must be processed in batches of 96 for the machine to run at full capacity. To maximize efficiency, tests were only run when batches were completed. The 30-day interval provided ample time to complete sample processing. During this period, unit nurses visited HPV-positive women at home to deliver their results. HPV-positive women were then scheduled to attend a triage center to undergo VAT and, if eligible, cryotherapy the same day. HPV-positive women who could not be reached were contacted at least two more times by a CHP and asked to make an appointment at a triage center to receive their results. HPV-negative women received their results at the 30-day appointment in their local health units and were recommended to be rescreened in 5 years. HPV-positive women who attended triage center appointments but were ineligible for ablation were referred to their regional hospital to undergo colposcopy and biopsy. Delivery of biopsy results and any subsequent procedures took place within the public health system but outside the context of CAPE.

Data collection instruments for screening and VAT were created by the MOH and BHI and are available online in El Salvador’s cervical cancer control guidelines.^24^ Colposcopy and biopsy information was recorded on standard MOH forms at hospitals. In phase I, BHI created and managed the only CAPE database while collaborating with the MOH to develop an online database that eventually became part of the El Salvador National Epidemiologic Surveillance System. In phases II and III, the CAPE online database was completed and management was transferred to the MOH. BHI received copies of data collection instruments and maintained an independent database that was used for all analyses presented here.

For comparisons across phases, we used Wilcoxon-type trend tests for HPV positivity rates, Pearson’s χ^2^ and *t* tests for sociodemographic variables and program indicators, and Kruskal-Wallis test for median number of days between procedures. The significance level was set at .05 for all tests. All statistical analyses were conducted using Stata 14.1 (StataCorp, College Station TX).

### Patients and Public Involvement Statement

Patients and the public were not involved in the design, conduct, reporting, or dissemination of this project.

## RESULTS

Results from CAPE phases I and II have been published previously.^19,20^ In phase III, 17,965 women were screened using careHPV (there are no data on women who were invited to participate but refused). The mean age for these participants was 41.4 years (standard deviation, 8.2 years) and the majority lived in rural areas (76.8%) versus urban areas (23.2%). Other demographic data collected in earlier phases were not collected during phase III. Available variables and differences found in the S&T cohorts across the three CAPE phases are listed in Table 1. In phase III, there was no effect of age, time since last screen, or area of residence on any of the loss to follow-up measures described below.

**TABLE 1:**
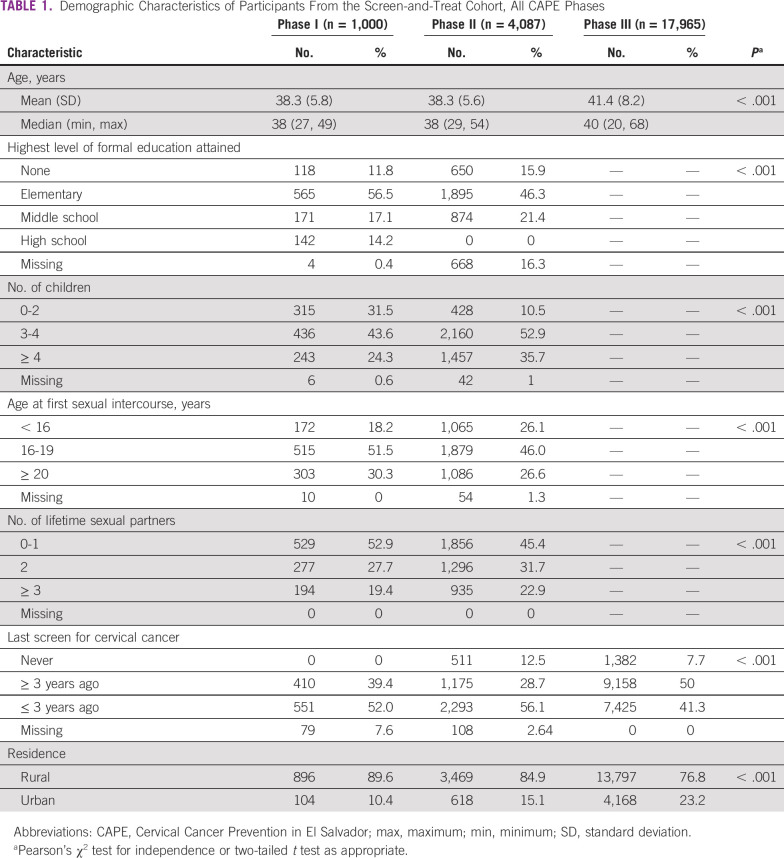
Demographic Characteristics of Participants From the Screen-and-Treat Cohort, All CAPE Phases

HPV positivity rates were 11.9% (119 of 1,000) in phase I, 11.4% (465 of 4,087) in phase II, and 12.3% (2,214 of 17,965) in phase III; these changes were not statistically significant (Wilcoxon-type trend test, *P* = .173).^25^ Age-adjusted HPV positivity rates also show little difference across the three phases (Table 2).

**TABLE 2:**
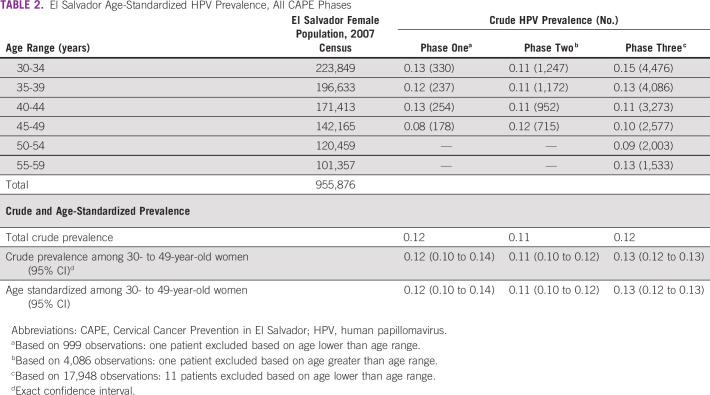
El Salvador Age-Standardized HPV Prevalence, All CAPE Phases

HPV-positive women were considered lost to follow-up (LTFU) if they did not complete indicated management (VAT and immediate cryotherapy or colposcopy, biopsy, and subsequent procedures) within 6 months. In phase III, 72.8% (1,612 of 2,214) of HPV-positive women completed VAT and cryotherapy within 6 months of screening (adherent), 4.6% (102 of 2,214) were classified as LTFU but returned and received VAT and cryotherapy after 6 months, 11.2% (248 of 2,214) were referred to colposcopy after VAT, and records for the remaining 11.4% (252 of 2,214) were missing.

Among women referred to colposcopy, 38.7% (96 of 248) attended the initial colposcopy appointment within 6 months, but treatment records were only available for 73 of these women. Of these, 19.0% (47 of 248) completed indicated treatment within 6 months, 6.5% (16/248) after 6 months, and 4.0% (10 of 248) received treatment but had no treatment date. We were unable to confirm if the remaining 70.5% (175 of 248) were LTFU or returned for treatment but their records were missing. Thus, in phase III, a total of 75% (1,659 of 2,214) of women completed their indicated procedures within 6 months of screening, 5.3% (118 of 2,214) after 6 months, and the remaining 19.7% (437 of 2,214) were either LTFU or had missing data. However, when combining missing data with women who completed treatment after 6 months, did not return for their HPV test results, and did not attend indicated procedures after referral, LTFU was 25.1% (555 of 2,214), which was significantly greater than phases I (6.7%) and II (7.7%; *P* > .001; Table 3).

**TABLE 3:**
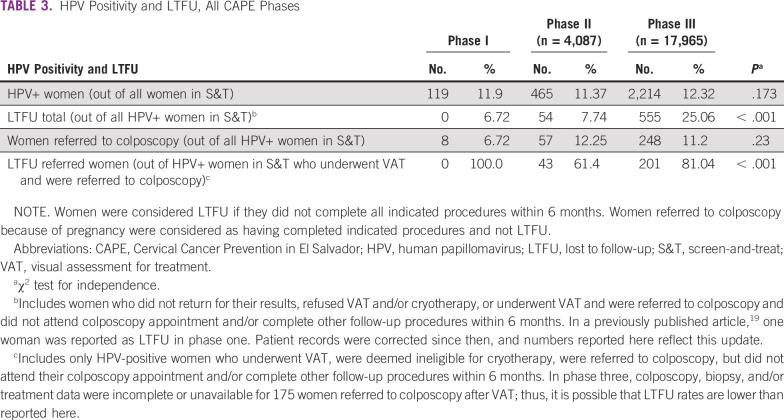
HPV Positivity and LTFU, All CAPE Phases

In phase III, reasons women were referred to colposcopy after VAT were: lesion suspicious for cancer (9 of 248, 3.63%), pregnancy (2 of 248, 0.81%), and unsatisfactory VAT (215 of 248, 86.69%). Data are missing for the remaining women (22 of 248, 8.87%). Among referred cases, biopsy results and treatment records were available for 84 of 248 (34.27%) and 73 of 248 (29.4%) women, respectively. Reasons for referral to colposcopy, biopsy results, and treatment types across phases are listed in Table 4.

**TABLE 4:**
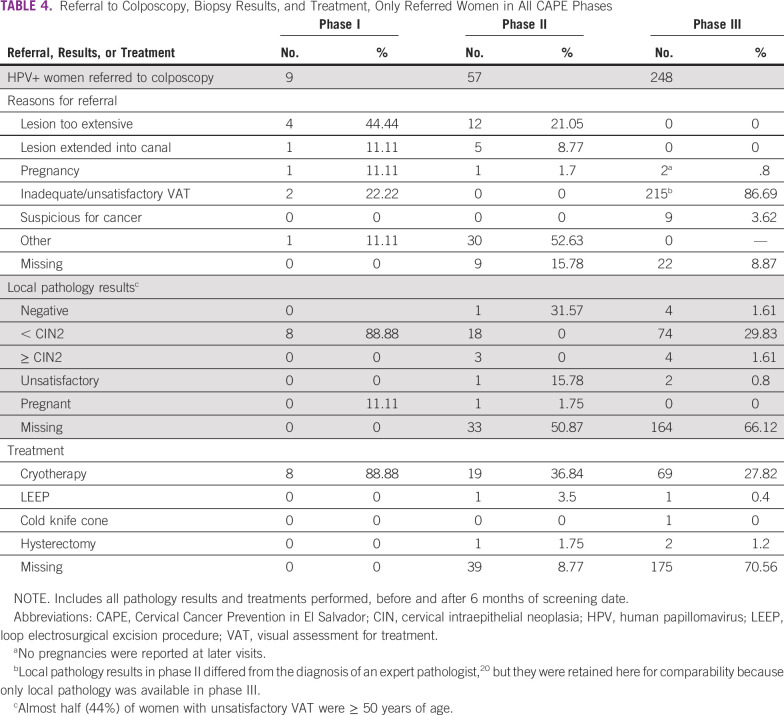
Referral to Colposcopy, Biopsy Results, and Treatment, Only Referred Women in All CAPE Phases

Delivery of HPV test results to all participating women was planned for 30 days after screening. As the project scaled up, the median day interval increased from 14 days (range, 1-379 days) in phase I to 41 days (range, 1-391 days) in phase II and 74 days (range, 1-761 days) in phase III. However, HPV-positive women in phase III received their results in fewer days than HPV-negative participants (80.3 *v* 102.8, *P* < .001). The median number of days between screening and colposcopy for referred patients decreased from phase I (137 days; range, 27-274 days) to phase III (98 days; range, 37-680 days; *P* < .001). The median number of days between colposcopy and treatment was also shorter in phase three than in phase II (18 days; range, 0-319 *v* 42 days; range, 12-163 days; *P* < .001; data for phase I were not available; Table 5).

**TABLE 5:**
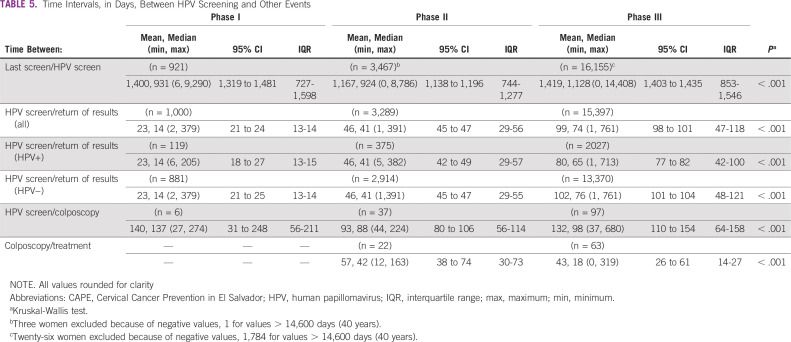
Time Intervals, in Days, Between HPV Screening and Other Events

## DISCUSSION

CAPE reached 28,050 underserved women in El Salvador and is the largest public-sector implementation program to date of an S&T protocol with a lower-cost HPV test.

As CAPE scaled up, there were increases in loss to follow-up rates. A study with nonattenders of conventional screening programs in the Paracentral region found that barriers to screening included embarrassment at being examined by a male doctor, misinformation about screening, and long wait times.^26^ High rates of migration may also play a role. Addressing such obstacles will be important to increase the future cost-effectiveness of the program.

An important limitation was inadequate data collection for women referred to colposcopy. In phase III, complete data for more than two-thirds of referred women were not available. Colposcopy and biopsy results came from the medical records of participating referral hospitals, and much of this information was not transferred to the electronic MOH database. Although it is impossible to determine if missing cases truly represent loss to follow-up, it is still the case that women referred to colposcopy face additional barriers to complete indicated procedures.

Another challenge was the long interval between screening and delivery of results to participants. This was partly due to the loss of two shipments of reagents for HPV test processing that occurred when cold-chain requirements were not followed correctly. Although this was eventually resolved, 562 samples were frozen at −20°C for approximately 6 months until analyses could resume. However, there were no differences in HPV positivity between these and the rest of the samples (12.39% *v* 12.32%, respectively; *P* = .962). Overall, HPV-positive women were prioritized and received their results earlier than those who tested HPV negative. The limited number of colposcopists also contributed to long wait times between appointments for referred women. Although mean number of days between procedures decreased with scale-up, high rates of missing data make it difficult to interpret this change. Table 6 provides a summary of obstacles that emerged during the implementation of the program.

**TABLE 6:**
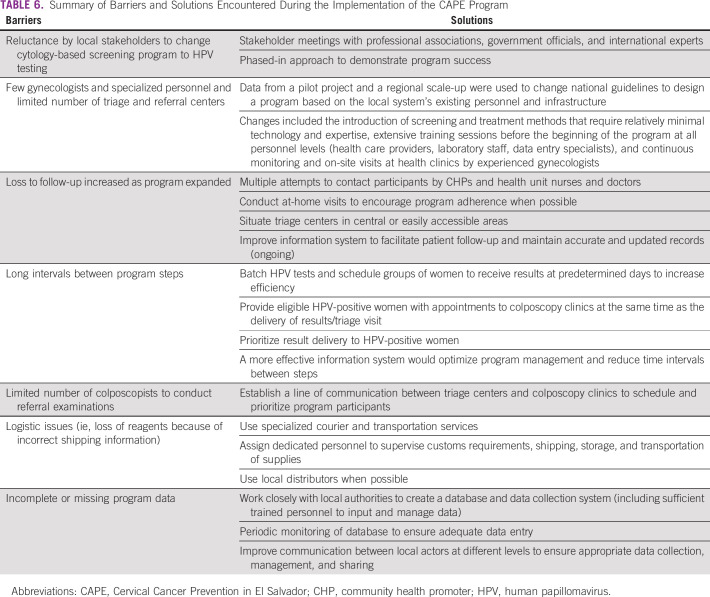
Summary of Barriers and Solutions Encountered During the Implementation of the CAPE Program

Despite these setbacks, CAPE demonstrated the potential of public-private sector collaborations in cervical cancer prevention efforts. The initial impetus for the project came from the possibility of obtaining the careHPV tests via an industry program. As a nonprofit organization and a government agency, respectively, BHI and the MOH had different strengths and capabilities. With approval from the MOH, BHI designed phase I as a pilot study, hired a physician (K.A.) to act as project coordinator, and supervised trainings for health care providers. The MOH provided the necessary personnel and infrastructure and, as CAPE expanded, took ownership of the program, while BHI served as technical advisor.

Another key element in the success of CAPE was the commitment of the national government and other stakeholders, including academic institutions, professional medical associations in El Salvador, the National Institutes of Health, and the Pan-American Health Organization. BHI and MOH representatives organized meetings and presentations with experts and local authorities and an early field visit to Nicaragua to observe a project using HPV testing. CAPE outcomes were presented at the end of each phase to government officials, which increased support for the project.

The lean model of CAPE, based on the efficient use of the MOH’s existing resources, helped transform it into a sustainable initiative. Findings from CAPE were instrumental in changing El Salvador cervical cancer guidelines to recommend HPV testing and S&T.^24^ Although data collection and management need improvement, the MOH now has a digital surveillance system for cervical cancer. Crucially, the MOH has now procured 160,000 lower-cost HPV tests to implement a national expansion of CAPE over the next 2 years. CAPE demonstrated that linking lower-cost HPV screening to a protocol that favors follow-up treatment over colposcopy and cytology can achieve high coverage levels in a low-resource setting. Similar ongoing projects in other Central American countries will provide additional data to inform the optimal deployment of this paradigm.
